# The critical role of *hcpR* in regulating nitrosative stress defense in *Clostridioides difficile*

**DOI:** 10.1128/aem.01988-25

**Published:** 2026-01-26

**Authors:** Sanjana Kalra, Toheeb O. Ayinde, Abiola O. Olaitan

**Affiliations:** 1 Department of Biology, University of Waterloo 8430, Waterloo, Ontario, Canada; University of Illinois Urbana-Champaign, Urbana, Illinois, USA

**Keywords:** short-chain fatty acid (SCFA), adaptation, toxins, *C. difficile*, stress defense, reactive nitrogen species (RNS), nitrosative stress

## Abstract

*Clostridioides difficile* is an anaerobic, toxin-producing pathogen that colonizes the host gastrointestinal tract. Within this hostile environment, the bacterium encounters stressors such as reactive nitrogen species (RNS), which impose nitrosative stress that must be mitigated for survival. This study aimed to elucidate the molecular mechanisms by which *C. difficile* defends against nitrosative stress. We screened an unordered transposon mutant library of the epidemic strain R20291 using a nitric oxide (NO) donor and identified a nitrosative stress-sensitive mutant with an inactivated *hcpR*, a transcriptional regulator of the Crp/Fnr family. Transcriptomic and metabolomic analyses were conducted, alongside the assessment of toxin production, a key virulence factor in *C. difficile*. Our results revealed that *hcpR* is critical for nitrosative stress adaptation, with the *hcpR*::Tn mutant displaying eightfold increased sensitivity to NO. The mutant also showed sensitivity to other RNS, but not to reactive oxygen species. RNA-seq analysis showed that *hcpR* regulates *hcp*, which is involved in NO detoxification, and *frdX*, an iron-sulfur binding protein. Knockdown of *hcp* and *frdX* individually conferred NO sensitivity similar to the *hcpR*-inactivated mutant. Kyoto Encyclopedia of Genes and Genomes pathway analysis of *hcpR*::Tn transcriptome revealed elevated expression of genes associated with butanoate metabolism. Furthermore, the *hcpR*::Tn strain showed increased TcdA/TcdB toxin levels compared to the wild type. Targeted metabolomics revealed that *hcpR* inactivation causes metabolic remodeling, shifting toward enhanced amino acid fermentation and increased short-chain fatty acid production, including butyrate. These findings demonstrate that *hcpR* is essential for nitrosative stress defense and contributes to virulence regulation through metabolic remodeling in *C. difficile*.

**IMPORTANCE:**

Within the host gastrointestinal tract, *Clostridioides difficile* encounters various toxic compounds, including reactive nitrogen species (RNS), which induce nitrosative stress. To survive in this hostile environment, the bacterium must mount an effective defense against these damaging agents. In this study, we identified the transcriptional regulator *hcpR* as a key factor in *C. difficile* ability to withstand nitrosative stress. Mutants lacking an intact *hcpR*, or the knockdown of its downstream targets *hcp* and *frdX*, showed increased sensitivity to RNS, confirming their roles in nitrosative stress adaptation. The *hcpR* mutant also produced significantly elevated levels of toxins (TcdA/TcdB), highlighting its influence on virulence. In addition, the mutant demonstrated significant metabolic changes, including increased production of short-chain fatty acids, such as butyrate, which is known to enhance toxin production. Together, these findings underscore *hcpR* as an important nitrosative stress defense regulator linking stress adaptation and virulence modulation through coordinated metabolic and transcriptional responses.

## INTRODUCTION

*Clostridioides difficile* is an anaerobic bacterial pathogen that colonizes the gastrointestinal tract of humans and animals, particularly the colon. It is a leading cause of healthcare-associated diarrhea, which can progress to colitis—a severe inflammation of the colon (1). Within the gut environment of the host, *C. difficile* is exposed to a range of stressors, including reactive compounds generated by the host immune response, such as reactive nitrogen species (RNS) and reactive oxygen species (ROS), as well as fluctuations in pH, oxygen levels, and nutrient availability (2–6). Among these, elevated RNS levels impose nitrosative stress (7), a challenge *C. difficile* must overcome for survival.

Pathogenic and environmental bacteria must adapt to chemically hostile conditions. Nitrosative stress, in particular, arises from increased levels of RNS like nitric oxide (NO), produced by host inducible NO synthase, and peroxynitrite (ONOO^−^), formed by the reaction of superoxide with NO (8). These reactive species disrupt essential cellular functions, causing DNA damage, lipid peroxidation, and ultimately cell death (9, 10). To counteract these effects, many bacteria have evolved specialized defense mechanisms (11–14). For example, *Escherichia coli* and *Salmonella* sp. express the *hmp* gene, which encodes flavohemoglobin that detoxifies NO by converting it to nitrate under aerobic conditions or to nitrous oxide (N_2_O) anaerobically (15, 16). Other systems include regulatory proteins such as NorR, a NO reductase regulator in *E. coli* (17), and NsrR, a NO-responsive transcriptional repressor found in species like *Streptomyces coelicolor* (18).

Another major nitrosative stress regulatory defense system is the HcpR transcription factor, which has been identified in both environmental and pathogenic anaerobes, such as *Desulfovibrio* spp. and *Porphyromonas gingivalis* (19–22). In these organisms, *hcpR* not only promotes bacterial survival under nitrosative stress but is also associated with enhanced virulence (20).

Studies have shown that *C. difficile* possesses stress defense response mechanisms that enable it to withstand a range of environmental challenges. One such mechanism involves the alternative sigma factor σ^B^ (*sigB*), a general stress regulator in Gram-positive bacteria. *sigB* modulates defenses against various stressors, including oxygen tension, RNS, ROS, acidification, cationic antimicrobial peptides, and antibiotic exposure (2, 23, 24). Despite these insights, the specific systems that *C. difficile* employs to combat RNS remain largely underexplored.

In this study, we identified *hcpR*, a transcriptional regulator in *C. difficile*, through genetic screening. Its inactivation increased sensitivity to nitrosative stress and caused significant transcriptional perturbations upon NO exposure. Functional analyses demonstrated that *hcpR* specifically mediates resistance to RNS, but not ROS. Furthermore, inactivation of *hcpR* led to elevated toxin production, accompanied by a metabolic shift characterized by increased levels of short-chain fatty acids (SCFAs), including butyrate. These findings indicate that *hcpR* plays a central role in nitrosative stress defense and metabolic remodeling, with potential impact on the modulations of *C. difficile* virulence factors.

## RESULTS

### *hcpR* protects *C. difficile* from nitrosative stress

**Fig 1:**
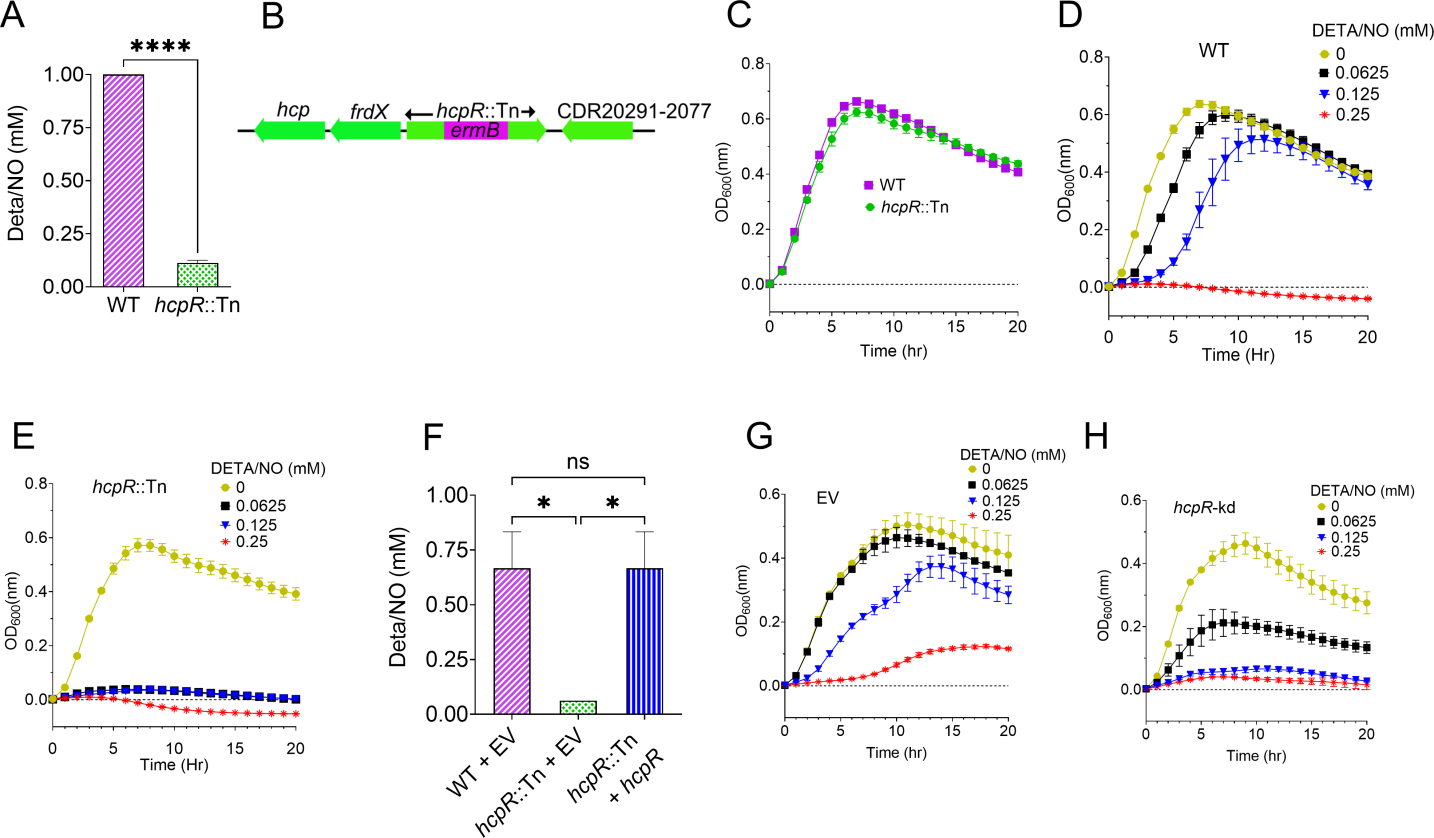
*hcpR* mediates protection against nitrosative stress in *C. difficile*. (**A**) MICs of DETA/NO, a NO donor, were determined for WT and *hcpR*::Tn strains in BHI after 24 hours. The *hcpR*::Tn mutant showed increased sensitivity (MIC = 0.125 mM) compared to the WT (MIC = 1 mM). ****: *P* < 0.0001 (two-tailed unpaired *t*-test). (**B**) Schematic representation of insertional inactivation of *hcpR* (*hcpR*::Tn). The mutant contains an *ermB* insertion that inactivates the *hcpR* coding sequence. Flanking genes are depicted, with arrows indicating their transcriptional orientation. (**C**) Growth curves of WT and *hcpR*::Tn strains in BHI without NO. Both strains showed comparable growth under non-nitrosative stress condition. (**D and E**) Growth of WT (**D**) and *hcpR*::Tn (**E**) strains in BHI with varying DETA/NO concentrations. WT tolerated up to 0.125 mM, whereas *hcpR*::Tn growth was inhibited at 0.0625 mM. Controls (0 mM) contained DMSO equivalent to that in 0.25 mM DETA/NO. (**F**) Complementation of *hcpR* restores nitrosative stress tolerance in *C. difficile*. MICs of DETA/NO were determined for the complemented strain (*hcpR*::Tn + *hcpR*), the *hcpR*::Tn mutant, and WT with EV (pRPF185). The mutant was highly sensitive to DETA/NO (MIC = 0.0625 mM), whereas complementation restored tolerance to WT levels (0.5–1 mM). ns: not significant; *: *P* < 0.05 (one-way analysis of variance with Tukey’s multiple comparison test). (**G and H**) Growth of WT EV (**G**) and *hcpR* CRISPRi knockdown; *hcpR*-kd (**H**) strains under nitrosative stress. WT EV tolerated 0.0625 mM DETA/NO, whereas *hcpR*-kd showed sensitivity or growth inhibition at the same concentration. All data are shown as mean ± SEM from three biological replicates.

We first assessed *C. difficile* tolerance to nitrosative stress by determining the minimum inhibitory concentration (MIC) of the NO donor diethylenetriamine/NONOate (DETA/NO) for the hypervirulent epidemic strain R20291 (25); the MIC was found to be 1 mM. Subsequently, in a screen of ~2,800 transposon (Tn) mutants from an unarrayed library derived from strain R20291 (26) to identify mutants sensitive or resistant to various stresses, including nitrosative stress, we found that most mutants exhibited only a modest twofold increase in sensitivity to DETA/NO. However, one mutant showed a striking eightfold increase in sensitivity, with an MIC of 0.125 mM compared to the R20291 wild-type (WT) MIC of 1 mM (Fig. 1A). Due to its significantly heightened sensitivity to the NO donor, we focused on this mutant. Whole-genome sequencing identified a single insertion of the *ermB* cassette in gene CDR20291_2076 at position 2,431,817 (corresponding to nucleotide 641 of 729 within the coding sequence) of the R20291 genome (FN545816.1), resulting in gene inactivation (Fig. 1B). CDR20291_2076 is annotated as a putative cNMP-binding regulatory protein, and domain analysis in NCBI shows that it belongs to the Crp/Fnr transcriptional regulator family. According to PaperBLAST and confirmed through BLAST searches in NCBI (27), the protein shows homology to HcpR from *P. gingivalis* (27% identity) and *Desulfovibrio vulgaris* (26% identity), as well as to HcpR2 from *Desulfovibrio desulfuricans* (34% identity). We found that CDR20291_2076 was indeed annotated as *hcpR* in RegPrecise (https://regprecise.lbl.gov/regulon.jsp?regulon_id=62803) along with a previous *in silico* analysis (28). We therefore reannotated CDR20291_2076 as *hcpR* and its Tn mutant as *hcpR*::Tn.

Growth curve assays showed that the WT and *hcpR*::Tn strains grew similarly under normal conditions without the NO donor (Fig. 1C). Under nitrosative stress, however, the WT strain tolerated DETA/NO concentrations up to 0.125 mM (Fig. 1D). In contrast, *hcpR*::Tn failed to grow even at 0.0625 mM (Fig. 1E), indicating that *hcpR* inactivation impairs NO tolerance. To confirm that the increased sensitivity to nitrosative stress was specifically due to *hcpR* inactivation and to rule out polar effect, we complemented the *hcpR*::Tn mutant with an intact *hcpR* gene under its native promoter. This complementation restored *hcpR*::Tn tolerance to NO donor, with the MIC returning to 1 mM, comparable to the WT empty vector (EV) strain (Fig. 1F). We further performed *hcpR* knockdown using CRISPR interference (CRISPRi). At 0.0625 mM DETA/NO, the WT EV control showed comparable growth to the dimethyl sulfoxide (DMSO) control (Fig. 1G). In contrast, a *hcpR* knockdown strain (*hcpR*-kd) exhibited impaired growth at the same concentration and failed to grow at 0.125 mM, which was tolerated by the WT EV (Fig. 1G and H). These results demonstrate that *hcpR* is required for *C. difficile* adaptation to nitrosative stress.

### Transcriptional regulation by *hcpR* during nitrosative stress

To investigate the regulatory role of *hcpR* in *C. difficile* under both unstressed and nitrosative stress conditions, we conducted transcriptome analysis comparing WT and *hcpR*::Tn strains. Under unstressed conditions, there were 24 significantly differentially expressed genes (DEGs) in the *hcpR*::Tn strain relative to WT, excluding the inactivated *hcpR* gene (Table 1). Several of the most upregulated genes were associated with butanoate (also called butyrate) metabolism according to Kyoto Encyclopedia of Genes and Genomes (KEGG) pathway analysis. The downregulated genes included *hcp*, CDR20291_2075 (reannotated as *frdX*, a ferredoxin-like iron-sulfur binding protein based on RegPrecise), and genes associated with fructose and mannose metabolism (Table 1). To identify potential direct targets of HcpR among these DEGs, we analyzed their promoter regions with the HcpR binding motif from RegPrecise using FIMO (MEME Suite). A putative binding site was identified only upstream of the *frdX-hcp* gene pair, which co-localizes with *hcpR*, suggesting that *hcpR* directly regulates these genes through a shared upstream promoter (29).

**TABLE 1:** Transcriptomic changes in *hcpR*::Tn compared to WT under identical untreated condition without NO exposure

Gene ID	Log_2_ fold change	Gene function	KEGG pathway
CDR20291_0963	−4.59	MBOAT family protein	Exopolysaccharide biosynthesis
CDR20291_0962	−3.96	AlgX/AlgJ SGNH hydrolase-like domain-containing protein	
CDR20291_2075 (*frdX*)	−3.04	Iron-sulfur binding protein	
*hcp*	−2.62	Hybrid-cluster protein	Nitrogen metabolism
CDR20291_3138	−1.34	Phosphotransferase system, IIc component	Phosphotransferase system/fructose and mannose metabolism
CDR20291_3137	−1.29	Phosphotransferase system, IId component	Phosphotransferase system
CDR20291_3139	−1.27	Phosphotransferase system, IIa component	
CDR20291_3136	−1.17	Phosphosugar isomerase	
CDR20291_3203	1.06	ABC transporter, ATP-binding protein	
CDR20291_1616	1.18	DUF917 domain-containing protein	
CDR20291_1617	1.18	Hydantoinase	
CDR20291_2234	1.33	LysR-family regulatory protein	
CDR20291_2076 (*hcpR*)	1.42	cNMP-binding regulatory protein	
CDR20291_2394	1.64	Histidinol-phosphate aminotransferase	
CDR20291_1615	1.69	Probable permease	
CDR20291_1747	2.10	HTH cro/C1-type domain-containing protein	
CDR20291_2931	3.86	Amino acid permease	
*abfH*	4.04	NAD-dependent 4-hydroxybutyrate dehydrogenase	Butanoate metabolism
*abfT*	4.26	4-Hydroxybutyrate CoA transferase	Butanoate metabolism
CDR20291_2229	4.29	Uncharacterized protein	
*abfD*	4.30	Gamma-aminobutyrate metabolism dehydratase/isomerase	Butanoate metabolism
CDR20291_2932	4.71	Glutamine amidotransferase	
*cat1*	5.25	Succinyl-CoA:coenzyme A transferase	Butanoate metabolism
*sucD*	5.28	Succinate-semialdehyde dehydrogenase [NAD(P)+]	Butanoate metabolism
CDR20291_2233	5.45	Membrane protein	

**Fig 2:**
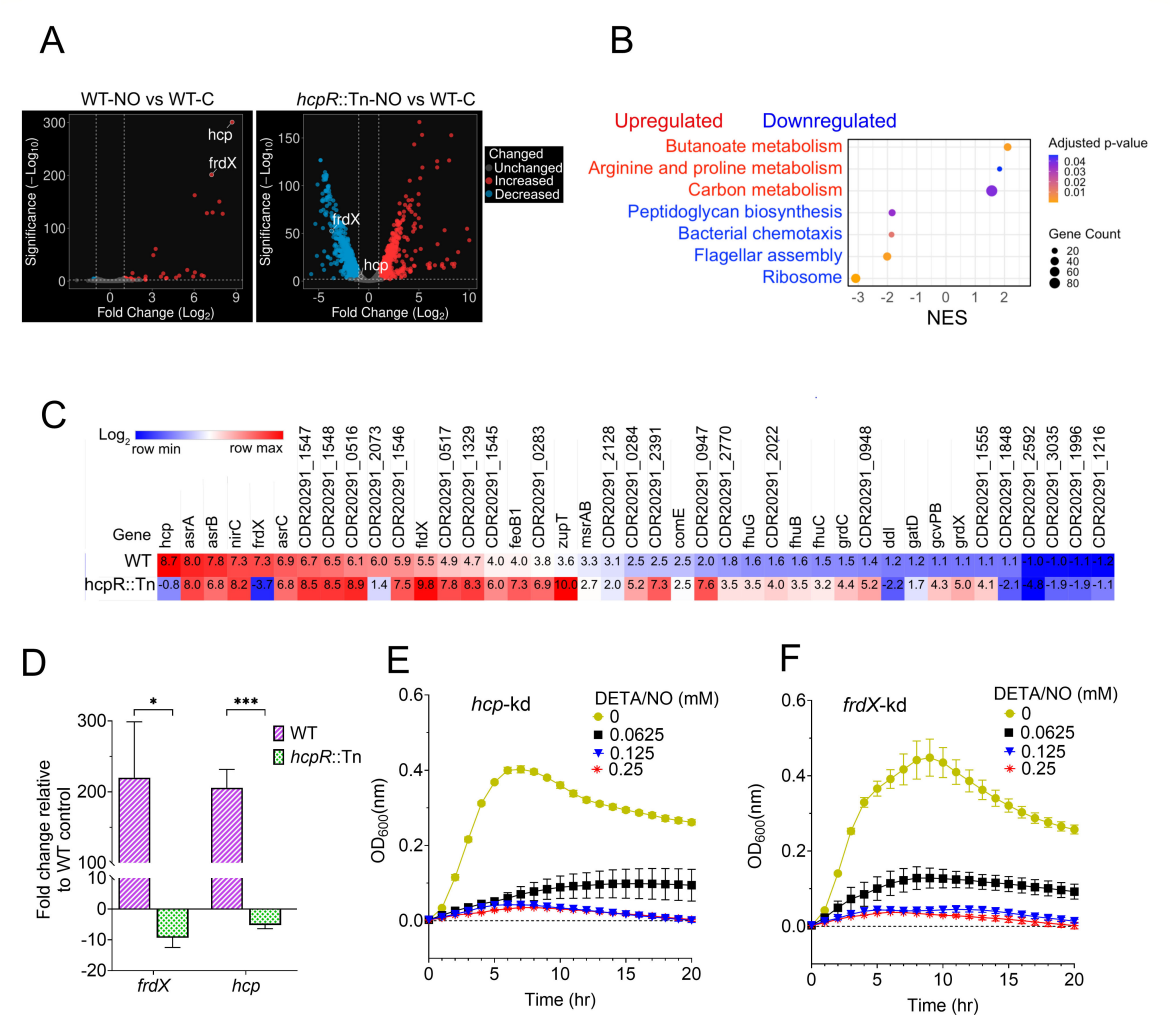
*hcpR*-mediated regulation of gene expression during nitrosative stress. (**A**) Volcano plots of DEGs in WT (left) and *hcpR*::Tn (right), both under nitrosative stress induced by exposure to DETA/NO at 0.0625 mM for 30 minutes. Expression changes are relative to WT untreated condition. Dashed lines indicate significance thresholds (log_2_ fold change ≥ 1 and FDR ≤ 0.01). NO exposure caused minimal transcriptional changes in WT, whereas *hcpR* inactivation led to extensive transcriptional perturbation. (**B**) GSEA with KEGG pathway enrichment in NO-treated *hcpR*::Tn relative to NO-treated WT. GSEA revealed pathways significantly enriched (adjusted *P* < 0.05) exclusively in the *hcpR*::Tn mutant under nitrosative stress. (**C**) Transcriptome profiles of DEGs shared between NO-treated WT and NO-treated *hcpR*::Tn strains, each relative to the untreated WT. The heatmap highlights differences in gene induction by nitrosative stress between strains with intact versus inactivated *hcpR*. Among these genes, *hcp* and *frdX*, known targets of *hcpR*, were induced in WT but not in *hcpR*::Tn. *hcp* was modestly downregulated in the *hcpR*::Tn strain (log_2_ fold change = –0.8; below the cutoff of ≥1) but was included for comparison with its expression in the WT. (**D**) Transcriptional analysis of *hcpR* targets (*hcp* and *frdX*) in NO-treated WT and NO-treated *hcpR*::Tn strains, each relative to the untreated control via qPCR. Both targets were strongly induced by NO in WT but downregulated under the same condition in *hcpR*::Tn. Expression was normalized to the 16S rRNA housekeeping gene. All data are presented as means ± SEM from four biological replicates. *: *P* < 0.05; ***: *P* ≤ 0.001 (two-tailed multiple unpaired *t*-test with Holm-Šídák multiple comparison test). (**E and F**) Growth of CRISPRi knockdown strains targeting *hcp* (**E**) and *frdX* (**F**) under varying concentrations of the NO donor DETA/NO. Both knockdowns were sensitive to nitrosative stress relative to the same strains grown in the DMSO control lacking the NO donor. All data are presented as means ± SEM from three biological replicates.

Under nitrosative stress, we assessed the role of *hcpR* by comparing the transcriptomes of NO-treated WT and NO-treated *hcpR*::Tn strains, each relative to untreated WT. The WT showed a modest response, with 42 DEGs (~1.2% of the genome; Fig. 2A; Table S1), whereas *hcpR*::Tn exhibited extensive transcriptional disruption, with 1,481 DEGs (~42% of the genome; Fig. 2A; Table S2). Comparison of NO-treated *hcpR*::Tn and NO-treated WT strains revealed that 1,350 genes were uniquely differentially expressed due to *hcpR* inactivation under nitrosative stress (Table S3). Gene set enrichment analysis (GSEA) of KEGG pathways using a stringent cutoff (p.adjust < 0.05) identified significant enrichment of multiple metabolic pathways. Upregulated genes were enriched in arginine/proline and carbon metabolism (excluding butanoate metabolism impacted mainly by *hcpR* inactivation). In contrast, downregulated genes were strongly enriched for ribosome function, flagellar assembly, bacterial chemotaxis, and peptidoglycan biosynthesis (Fig. 2B). Using a relaxed threshold (*q* ≤ 0.25) revealed additional enriched pathways, including glycerophospholipid and nicotinate/nicotinamide metabolism among upregulated genes, and amino acid and fatty acid biosynthesis pathways among downregulated genes (Table S4). These results indicate that *hcpR* inactivation broadly impacts both metabolic and structural processes during nitrosative stress.

Among the genes shared between NO-treated *hcpR*::Tn and NO-treated WT strains (Table S5), only *hcp* and *frdX* were identified as direct targets of *hcpR*. Both genes were strongly induced under nitrosative stress in the WT strain but remained uninduced in the *hcpR*::Tn mutant (Fig. 2C). Quantitative PCR (qPCR) confirmed this trend; in the WT, *hcp* and *frdX* were upregulated by ~206 ± 26-fold and 220 ± 79-fold, respectively. In contrast, their expression in the mutant was reduced by about ~5 ± 1fold and 9 ± 3-fold, respectively (Fig. 2D). To investigate the role of the *frdX-hcp* gene cluster in nitrosative stress defense, we individually knocked down *hcp* and *frdX*. Under nitrosative stress, both knockdown strains showed impaired growth compared to unstressed controls (Fig. 2E and F). The negative control, knockdown *nimB*, a gene not known to contribute to NO detoxification under the NO levels used in this study, was not impacted by NO exposure (Fig. S1). Overall, these findings demonstrate that *hcpR* inactivation leads to extensive transcriptional dysregulation under nitrosative stress and that it activates the *frdX-hcp* cluster in response to NO.

### *hcpR* protects against nitrosative stress but not oxidative stress

**Fig 3:**
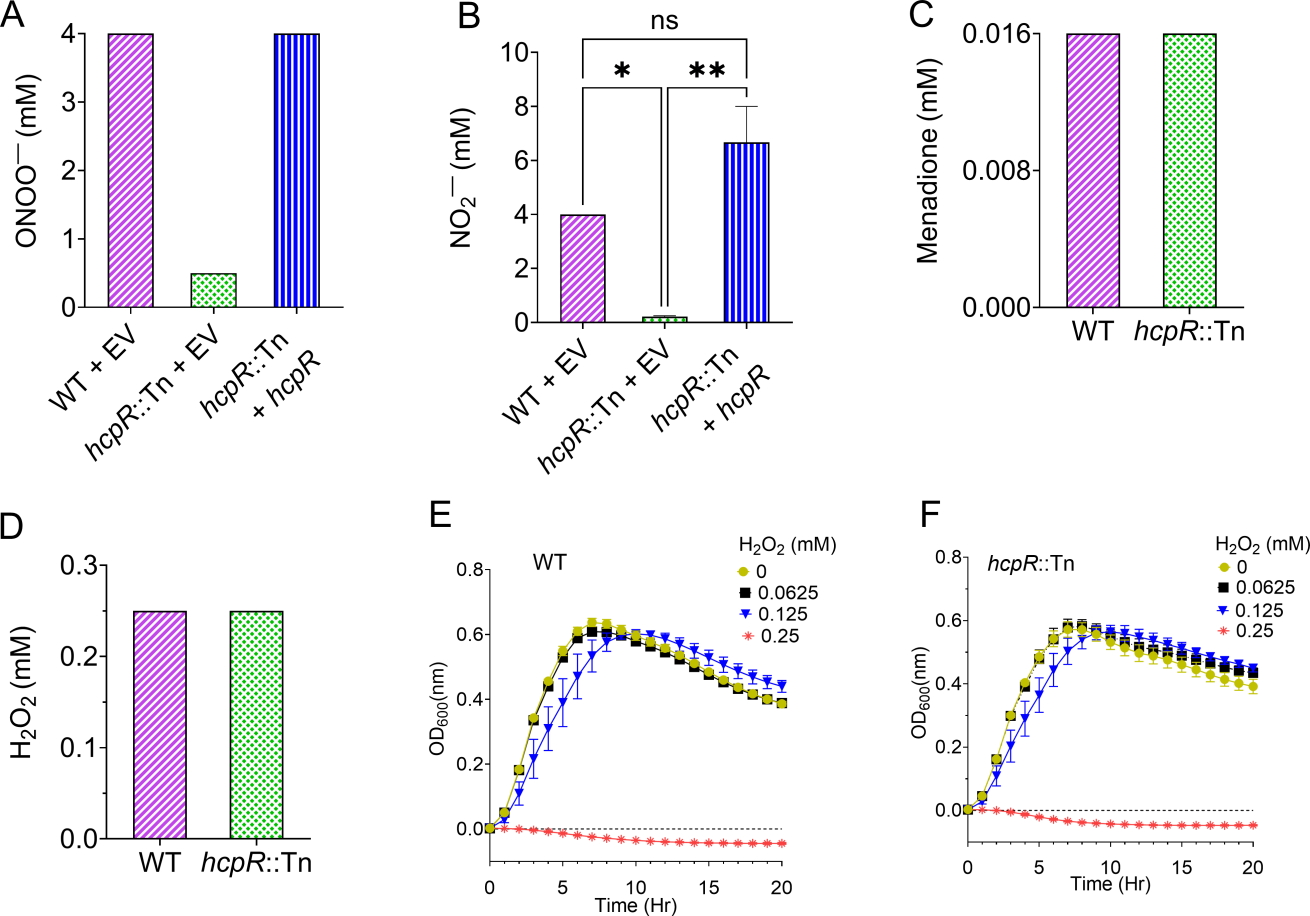
*hcpR* specifically defends against nitrosative stress and does not provide protection against oxidative stress. (**A and B**) MICs of ONOO^−^ (A) and NO_2_^−^ (B) were determined for WT EV, *hcpR*::Tn EV, and the complemented strain (*hcpR*::Tn + *hcpR*). The *hcpR*::Tn mutant was sensitive to both RNS sources, whereas complementation restored tolerance to the same level as WT EV. ns: not significant; *: *P* < 0.05; **: *P* ≤ 0.01 (one-way analysis of variance with Tukey’s multiple comparison test). (**C and D**) MICs of menadione (**C**) and H_2_O_2_ (**D**) were determined for WT and *hcpR*::Tn. Both strains exhibited similar tolerance to these ROS. (**E and F**) Growth curves of WT (**E**) and *hcpR*::Tn (**F**) under oxidative stress with varying concentrations of H_2_O_2_ (0.0625–0.25 mM). Strains were grown in BHI supplemented with H_2_O_2_ and untreated control. Both strains displayed similar growth patterns, tolerating 0.125 mM H_2_O_2_ or being inhibited at 0.25 mM. All data are presented as means ± SEM from three biological replicates.

Building on the observed sensitivity of the *hcpR*::Tn mutant to NO, we examined its response to other sources of nitrosative stress. Specifically, *hcpR*::Tn exhibited an eightfold increase in sensitivity to peroxynitrite (ONOO^−^), with an MIC of 0.5 mM compared to 4 mM for the WT strain (Fig. 3A). Complementation of *hcpR* restored the mutant’s tolerance, increasing the MIC to 2 mM. The mutant also exhibited a 16- to 32-fold increase in sensitivity to nitrite (NO_2_^−^), with MIC values ranging from 0.125 to 0.25 mM, while the WT strain maintained an MIC of 4 mM (Fig. 3B). Complementation with intact *hcpR* similarly reversed this sensitivity, restoring MIC values to 4–8 mM, similar to the WT. To determine whether *hcpR* contributes to oxidative stress defense, WT and *hcpR*::Tn strains were exposed to ROS, including menadione (a superoxide generator) and hydrogen peroxide (H_2_O_2_), which produces hydroxyl radicals (30). Both strains showed similar sensitivity to menadione and H_2_O_2_, with MICs of ~0.0156 and 0.25 mM, respectively (Fig. 3C and D). Growth assays under H_2_O_2_-induced stress confirmed that both strains experienced identical growth inhibition at 0.25 mM, with growth resuming below this concentration (Fig. 3E and F). Taken together, these results reveal that *hcpR* specifically mediates in nitrosative stress defense in *C. difficile* but does not modulate defense against oxidative stress.

### *hcpR* does not mediate defense against MTZ-induced stress

**Fig 4:**
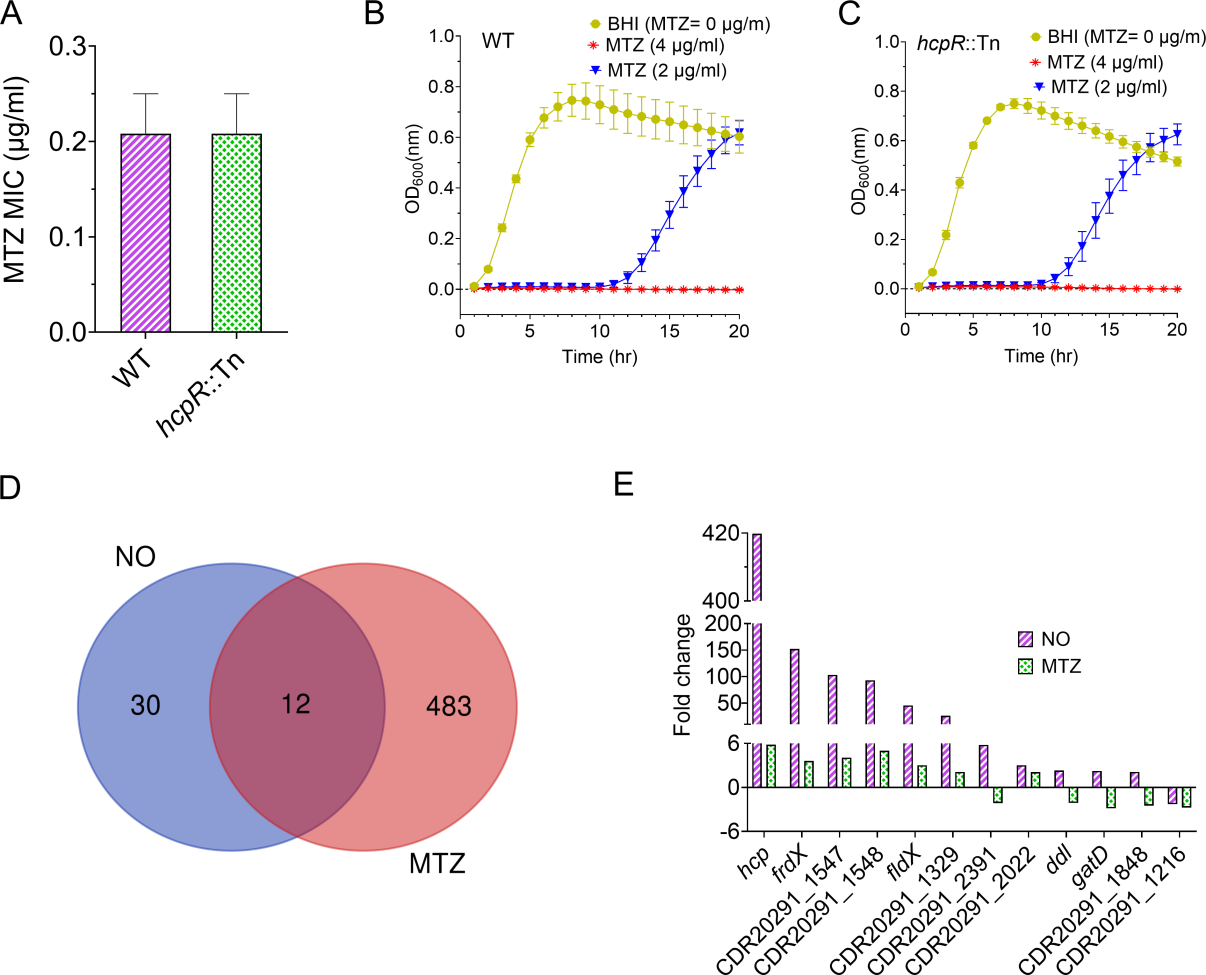
*hcpR* does not provide protection against MTZ. (**A**) The MIC of MTZ, a nitroheterocycle-based antibiotic, was determined for WT and *hcpR*::Tn strains. Both strains exhibited the same level of sensitivity to MTZ, with an MIC of 0.25 µg/mL. (**B and C**) Growth curves of WT (**B**) and *hcpR*::Tn (**C**) in response to MTZ. Strains at exponential phase were grown in BHI supplemented with varying concentrations of MTZ and BHI containing DMSO as the control. Both strains exhibited similar growth response to MTZ. All data are presented as means ± SEM from three biological replicates. (**D**) Venn diagram showing the transcriptomes of significantly DEGs in *C. difficile* R20291 exposed to NO or MTZ for 30 minutes, each relative to the DMSO control. The intersection shows that only 12 genes were shared between the two stressors. (**E**) Comparison of the 12 DEGs shared between the transcriptomes of NO- and MTZ-treated *C. difficile* R20291. NO induced stronger upregulation of certain genes, including the *hcpR* targets (*hcp* and *frdX*), compared to MTZ.

Metronidazole (MTZ), a nitroheterocyclic antibiotic effective against *C. difficile*, is believed to exert its bactericidal effect by generating cytotoxic nitro radical intermediates that mimic nitrosative stress (31, 32). To investigate whether *hcpR* influences MTZ sensitivity, we compared the susceptibility of WT and *hcpR*::Tn strains to MTZ. Both strains showed similar MICs of 0.125–0.25 µg/mL (Fig. 4A). Growth assays showed similar response, with both strains inhibited at 4 µg/mL MTZ and reduced growth at lower concentration (Fig. 4B and C). To further explore the relationship between MTZ and NO-mediated nitrosative stress defenses, we analyzed previously published RNA-seq data (26) comparing transcriptomes of MTZ-treated and NO-treated WT *C. difficile* R20291. MTZ exposure resulted in 495 DEGs, whereas NO exposure affected only 42 DEGs. Twelve DEGs were shared between the two treatments (Fig. 4D). Furthermore, *hcp* and *frdX*, known *hcpR* targets, were among the most strongly upregulated genes by NO (~420- and 152-fold, respectively) but were only modestly induced by MTZ (~6- and 4-fold, respectively; Fig. 4E). These findings suggest that *hcpR* is not essential for protection against MTZ-induced toxicity or its associated nitro radical intermediates in *C. difficile*.

### *hcpR* modulates *C. difficile* toxin production

**Fig 5:**
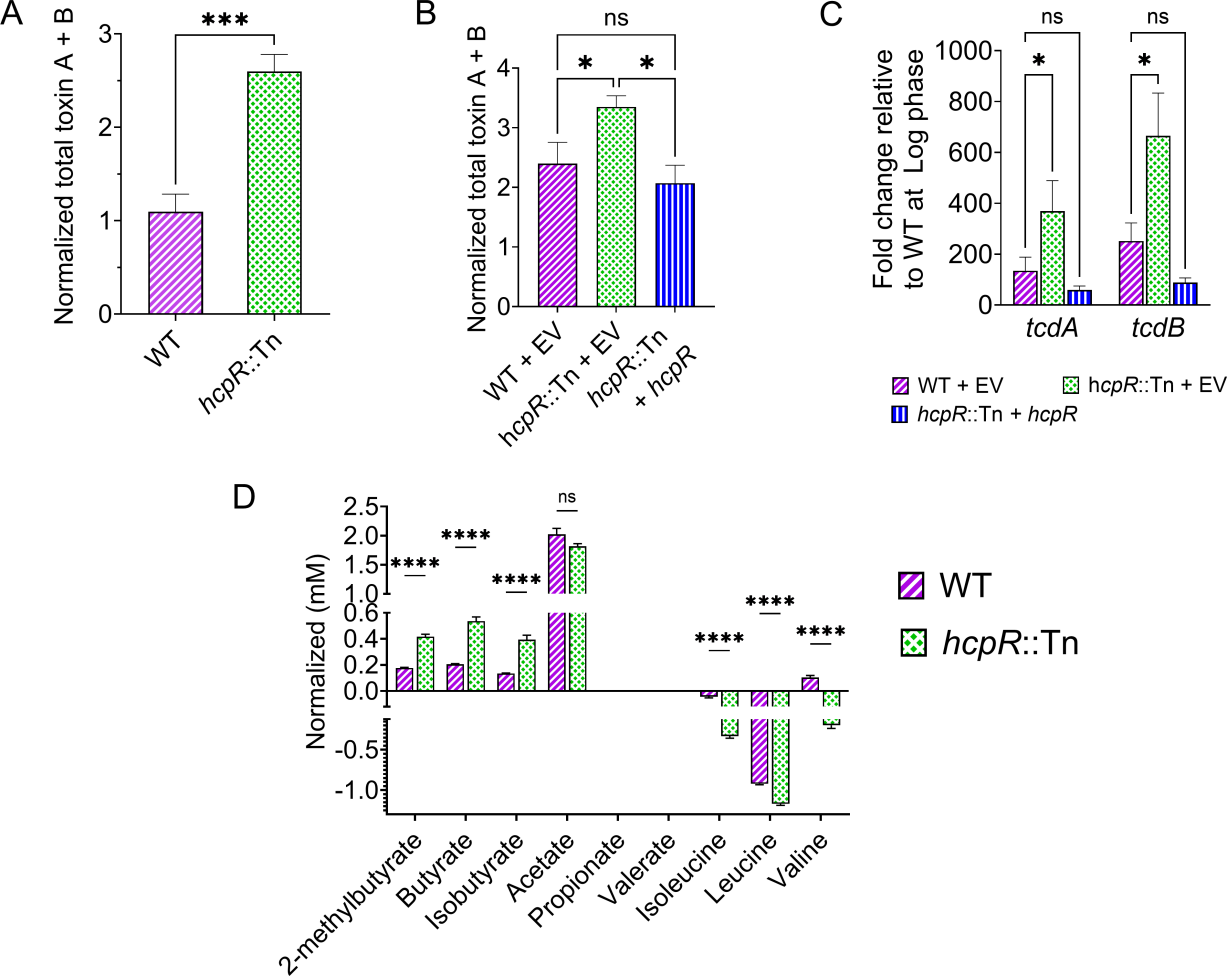
Impact of *hcpR* on toxin production. (**A**) Quantification of toxins (TcdA/TcdB) in WT and *hcpR*::Tn strains revealed that the mutant produced higher levels of toxin compared to WT. Data are presented as means ± SEM from six biological replicates in technical duplicates. ***: *P* ≤ 0.001 (two-tailed unpaired *t*-test). (**B**) Quantification of toxins (TcdA/TcdB) in WT EV, *hcpR*::Tn EV, and complemented *hcpR*::Tn (*hcpR*::Tn + *hcpR*) strains. The *hcpR*::Tn EV produced higher toxin levels than WT EV, whereas complementation of the mutant with intact *hcpR* restored toxin production to WT levels. Data are presented as means ± SEM from eight biological replicates in technical duplicates. ns: not significant; *: *P* < 0.05 (one-way analysis of variance with Tukey’s multiple comparison test). (**C**) qPCR quantification of *tcdA* and *tcdB* expression levels in WT EV, *hcpR*::Tn EV, and *hcpR*::Tn + *hcpR* strains. Overnight cultures were grown to exponential phase (OD_600_ = 0.3) and to stationary phase (24 hours) for gene expression. The *hcpR*::Tn EV strain exhibited higher expression of toxin genes compared to WT EV, whereas complementation of the mutant with intact *hcpR* restored expression to WT levels. Gene expression levels are shown relative to WT EV at exponential/log phase. Data are presented as means ± SEM from five biological replicates. ns: not significant; *: *P* < 0.05 (two-way analysis of variance with Tukey’s multiple comparison test). (**D**) Targeted metabolomics of WT and *hcpR*::Tn strains using ^1^H-NMR. The *hcpR*::Tn mutant exhibited increased levels of SCFAs and reduced BCAAs compared to WT. Acetate and propionate were not detected by ^1^H-NMR, which may indicate their absence or concentrations below the detection limit. Data are presented as means ± SEM from six biological replicates. ns: not significant; ****: *P* ≤ 0.0001 (two-tailed multiple unpaired *t*-test with Holm-Šídák multiple comparison test).

Nitrosative stress defense mechanisms have been linked to bacterial virulence (33). To assess the impact of *hcpR* inactivation on toxin virulence factor, we quantified toxin production. The *hcpR*::Tn mutant produced ~2.4-fold higher levels of toxins (TcdA/TcdB) compared to the WT strain (Fig. 5A). To further validate this observation, we examined toxin levels in the *hcpR*::Tn strain carrying either an EV or a complementation construct. The *hcpR*::Tn with an EV maintained elevated toxin production (Fig. 5B), whereas introduction of an intact *hcpR* gene restored toxin levels to those observed in the WT EV strain. We further quantified the expression levels of *tcdA* and *tcdB* toxin genes using qPCR. In the *hcpR*::Tn EV strain, expression of both *tcdA* and *tcdB* was significantly elevated compared to the WT EV strain. In contrast, these elevated expression levels were restored to WT EV levels in the *hcpR*::Tn + *hcpR*-complemented strain (Fig. 5C).

Given that genes involved in butanoate metabolism were upregulated in the *hcpR*::Tn transcriptome (Table 1) and considering that increased butyrate levels are known to enhance toxin production (34, 35), we investigated whether SCFA metabolites were elevated in the mutant strain. Targeted metabolomics confirmed increased levels of SCFAs, including butyrate, isobutyrate, and 2-methylbutyrate, in the *hcpR*::Tn mutant. Concurrently, levels of branched-chain amino acids (BCAAs), such as valine, leucine, and isoleucine were reduced (Fig. 5D). Overall, these findings indicate that *hcpR* impacts *C. difficile* toxin production and contributes to metabolic changes affecting SCFA levels.

## DISCUSSION

In the host gastrointestinal tract, *C. difficile* is exposed to nitrosative stress caused by elevated RNS, which are harmful and must be counteracted for bacterial survival (7). In this study, we demonstrated that the inactivation of *hcpR* (*hcpR*::Tn) increased *C. difficile* susceptibility to multiple RNS, including NO, ONOO^−^, and NO_2_^−^, a related nitrogen metabolite known to contribute to RNS generation. These findings align with previous reports of HcpR-mediated nitrosative stress defense in other bacteria, such as *Desulfovibrio* spp. and *P. gingivalis*, which inhabit environmental and oral niches, respectively (19, 20). Inactivation of *hcpR* rendered these bacteria sensitive to nitrosative stress in the presence of elevated nitrite or S-nitrosoglutathione. Together, these results paint a picture that *hcpR*-mediated defense against nitrosative stress is a conserved survival strategy across both diverse bacterial species and ecological niches. Our data showed that *hcpR* specifically confers protection against RNS, but not ROS, consistent with observations in *P. gingivalis* (20). This functional specificity might be explained by the fact that *C. difficile* encodes dedicated ROS defense systems, including the peroxide stress regulator PerR and various oxidative stress detoxification enzymes (36, 37), which likely operate independently of RNS-responsive pathways. This indicates that *C. difficile* uses distinct regulatory systems to manage different stresses in the host environment.

Our findings show that inactivation of *hcpR* markedly reshapes the global transcriptional landscape of *C. difficile* under nitrosative stress and demonstrated that *hcpR* is essential for maintaining transcriptional homeostasis during nitrosative stress, protecting both metabolic and structural processes from widespread dysregulation. Under nitrosative stress, the *hcpR*::Tn mutant exhibited increased amino acid catabolism and central carbon metabolism, reflecting an attempt to increase energy production under stress. Concurrent downregulation of energy-intensive processes, including ribosome biogenesis and cell wall synthesis, indicates a shift toward energy conservation. Together, these changes provide insight into the stress-adaptive metabolic reprogramming driven by *hcpR* inactivation.

We found that *hcpR* regulates the expression of *hcp* and *frdX* in response to nitrosative stress. These genes are located directly upstream of *hcpR* (Fig. 1B), a genomic arrangement similar to that seen in *Desulfovibrio* spp., such as *D. vulgaris* Hildenborough and *Desulfovibrio alaskensis* G20, although with some variation in gene content (29, 38). The distinct but coordinated expression patterns of *hcp* and *frdX* in WT and *hcpR*::Tn strains, together with a shared HcpR binding site and similar nitrosative stress–responsive phenotypes, support their co-regulation by *hcpR* in *C. difficile*. This pattern is consistent with findings in *D. vulgaris* Hildenborough (39).

The *hcp* gene is a well-established HcpR target that plays a key role in NO detoxification (40). In *P. gingivalis*, *hcp* is the primary HcpR-regulated gene and is essential for nitrosative stress adaptation and intracellular survival (41). In *E. coli*, deletion of *hcp* increases NO sensitivity and impairs its reductive detoxification to nitrous oxide (N_2_O) (42). Similarly, in *D. vulgaris*, N_2_O binds Hcp, reinforcing its role in NO detoxification (43). Consistent with these findings, we showed that knockdown of *hcp* in *C. difficile* increased sensitivity to NO. Although our data suggest that *frdX* also contributes to nitrosative stress adaptation, its exact function remains unclear. *frdX* encodes a ferredoxin-like iron-sulfur binding protein typically involved in electron transfer via Fe-S clusters (28, 44). It is plausible that *frdX* participates in redox regulation or electron transfer during *hcpR*-mediated stress defense responses (29).

Our observation that the *hcpR* mutant displayed similar susceptibility to MTZ suggests that *hcpR* is unlikely to play a major role in the bacterial defense against MTZ, which is thought to induce nitrosative stress. Transcriptomic analysis showed that MTZ exposure elicited a broader and more extensive transcriptional response than NO exposure in the WT, with distinct expression profiles. Additionally, the *hcpR* target genes *hcp* and *frdX* were strongly induced by NO but only modestly upregulated by MTZ. These findings suggest that MTZ and NO elicit separate or unique regulatory pathways to deal with these stressors. The cellular response to MTZ detoxification likely involves mechanisms that are largely independent of *hcpR*. This is intriguing given that MTZ is believed to generate reactive nitro radicals capable of inducing nitrosative stress (32).

Beyond its role in stress defense, our findings indicate that *hcpR* impacts a key virulence factor in *C. difficile*. The *hcpR*::Tn mutant showed increased production of toxins (TcdA/TcdB), including at the transcriptional level. This virulence factor is central to *C. difficile* pathogenesis and contributes to inflammation of the colon (45). The increased expression of butanoate (butyrate) metabolism genes in *hcpR*::Tn correlates with elevated butyrate levels. Additionally, we observed increased concentrations of other SCFAs and decreased levels of BCAAs, suggesting enhanced BCAA catabolism. Given that BCAAs such as valine and isoleucine are precursors for SCFAs like isobutyrate and 2-methylbutyrate, respectively (46), this pattern indicates that *hcpR* inactivation triggered a metabolic shift toward increased amino acid fermentation. Although our analysis focused on targeted metabolites, we observed additional metabolic changes beyond SCFAs between WT and *hcpR*::Tn strains, including differences in 3-phenylpropionate, isocaproate, and trehalose (Table S6). These findings point to broader metabolic remodeling resulting from the loss of a functional *hcpR*. The overall metabolic shift may serve as a compensatory adaptive mechanism to support energy production and maintain redox balance in the stress-sensitive mutant. Butyrate and other SCFAs are known to enhance toxin production in *C. difficile* (34, 35, 47), which may explain the elevated toxin levels observed in the *hcpR*::Tn mutant. The increased toxin production is likely a secondary consequence of the metabolic reprogramming advantages conferred by *hcpR* inactivation, rather than a direct effect of *hcpR* loss. Indeed, among the DEGs in the *hcpR*::Tn mutant, only the *frdX-hcp* cluster contains a putative HcpR binding site, suggesting that *hcpR* does not directly regulate butyrate or other SCFA biosynthesis. Hence, the upregulation of butanoate metabolism likely reflects broader metabolic changes triggered by increased nitrosative stress sensitivity.

In summary, our study demonstrates that *hcpR* plays a key role in nitrosative stress defense in *C. difficile*. Inactivation of *hcpR* compromises tolerance to RNS and leads to widespread transcriptional and metabolic changes, including increased SCFAs and elevated toxins. These findings highlight *hcpR* as a crucial transcriptional regulator that contributes to stress adaptation and also modulates virulence-related processes.

## MATERIALS AND METHODS

### MIC assay and Tn mutant screening

*C. difficile* strains were cultured in pre-reduced Brain Heart Infusion (BHI) medium, supplemented with 1.5% agar when required, and incubated overnight at 37°C in a Whitley A35 anaerobic workstation (Don Whitley Scientific) under a gas mixture of 85% N_2_, 5% CO_2_, and 10% hydrogen. An unordered Tn-mutant library of *C. difficile* R20291, previously generated using plasmid pRPF215 (26, 48), was used for nitrosative stress screening. Briefly, an overnight culture of R20291 carrying pRPF215, a Himar1 mariner delivery vector, was subcultured and grown to an OD_600_ of 0.2 in the absence of thiamphenicol. Himar1 transposition was induced with 100 ng/mL anhydrotetracycline and supplemented with 20 µg/mL lincomycin, followed by overnight incubation. The following day, several serial dilutions of the culture containing Tn mutants were plated on pre-reduced BHI agar supplemented with 20 µg/mL lincomycin. After incubation, individual Tn colonies were picked into 96-deep-well plates containing BHI with 20 µg/mL lincomycin and incubated. The unordered Tn-mutant library was subsequently stored for screening. Tn mutants were maintained on BHI agar containing 20 µg/mL lincomycin, and spore stocks were revived on BHI supplemented with 0.1% taurocholate, 250 µg/mL cycloserine, and 8 µg/mL cefoxitin. Screening of the Tn-mutant library was performed using overnight cultures grown in BHI with lincomycin and exposed to 1/4× and 1× MIC concentrations of test compounds in liquid BHI supplemented with 0.003% neutral red. The NO-sensitive mutant was whole-genome sequenced to identify the gene disrupted by *ermB*. Sequencing was performed at SeqCenter, LLC, in Pittsburgh, USA. MICs of test compounds were determined using broth dilution in BHI with 0.003% neutral red. Doubling dilutions were prepared in 196 µL volumes in microplate wells, pre-reduced for 3 hours in an anaerobic chamber, and inoculated with 4 µL of overnight cultures. MICs were recorded after 24 hours of incubation. Compounds tested included RNS sources, such as DETA/NO (Cayman Chemical, catalog no. 82120), peroxynitrite (Calbiochem, catalog no. 516620), and sodium nitrite (Sigma Aldrich, catalog no. S2252), and ROS sources including menadione (Sigma Aldrich, catalog no. M5625) and H_2_O_2_ (ThermoFisher Scientific, catalog no. H325500). MTZ (Sigma Aldrich, catalog no. M3761) MIC was determined using the agar dilution method as previously described (26). Stock solutions of all compounds were prepared in DMSO (Bioshop, Canada, catalog no. DMS555). All strains and plasmids used in this study are listed in Table S7.

### Growth curve assay

Doubling dilutions of test compounds (DETA/NO, H_2_O_2_, and MTZ) were prepared in 96-well microplates containing 100 µL of BHI broth, followed by a 3-hour pre-reduction in an anaerobic chamber. Overnight cultures of WT and *hcpR*::Tn strains were grown to early exponential phase (OD_600_ = 0.2–0.3, or back-diluted to 0.1 for MTZ testing). Each well was inoculated with 100 µL of culture, bringing the final volume to 200 µL. Plates were sealed with ELISA Plate Sealers (R&D Systems, Cat. No. DY992), and growth was monitored by measuring absorbance at 600 nm every 30 minutes over 24 hours using an Alto portable plate reader (Cerillo, USA) inside the anaerobic chamber at 37°C. OD_600_ readings were normalized to zero at the start of the growth curve. All experiments were performed in three technical and three biological replicates.

### Gene complementation

To complement *hcpR*::Tn, a 500 bp upstream region containing the native *hcpR* promoter along with the *hcpR* coding sequence from strain R20291 was PCR-amplified and cloned between the KpnI and BamHI sites of the pRPF185 plasmid (49). The plasmid was first introduced into electrocompetent *E. coli* SD46 (50) and then transferred to *hcpR*::Tn via conjugation. Briefly, 1 mL of overnight *E. coli* SD46 culture carrying the plasmid was pelleted and washed twice with phosphate-buffered saline. A 200 µL overnight culture of *hcpR*::Tn was added to the pellet, and the mixture was spotted onto BHI agar and incubated overnight. The next day, cells were harvested into 500 µL BHI broth and mixed, and 100 µL was spread on BHI agar containing 8 µg/mL cefoxitin, 250 µg/mL cycloserine, 15 µg/mL thiamphenicol, and 20 µg/mL lincomycin to select for transconjugants. After 2–3 days, colonies were transferred to fresh selective BHI agar for purification. An EV control (pRPF185) was also conjugated into *hcpR*::Tn and WT strains (no lincomycin added). Sensitivity assays for RNS were performed as already described. All primers used are listed in Table S8.

### Gene knockdown

Gene knockdown was carried out using CRISPRi. Two guide RNAs targeting *hcpR*, *frdX*, and *hcp* were synthesized by Bio Basic Inc. (Ontario, Canada) and cloned into the PmeI site of the pXWxyl-dcas9 vector (51). The resulting plasmids were conjugated into *C. difficile* strain R20291. Gene silencing was induced by culturing the bacteria in 2% xylose (BioShop Canada Inc., Cat. No. XYL001.500) and 15 µg/mL thiamphenicol, both during overnight growth and during subsequent growth to exponential phase prior to performing growth curve assays.

### Toxin quantification

WT and *hcpR*::Tn strains were cultured overnight on BHI agar and then grown in BHI broth for 24 hours. Cultures were centrifuged at 5,000 × *g* for 5 minutes at 4°C, and supernatants were stored at −80°C until toxin analysis. Toxins A and B were simultaneously quantified using an ELISA kit (tgcBIOMICS, Cat. No. TGC-E001-1) following the manufacturer’s instructions. Toxin levels were normalized to total protein content, measured using the Pierce BCA Protein Assay Kit (Thermo Scientific, Cat. No. LSG23227). For the *hcpR*-complemented strain and EV controls, a similar method was followed, except that cultures were grown in BHI supplemented with 2 µg/mL thiamphenicol to minimize the impact of the antibiotic on toxin production.

### ^1^H-NMR -based metabolomics

A portion of the supernatant used for toxin quantification was filtered through a 0.2 µm membrane filter. ^1^H nuclear magnetic resonance (NMR) experiments were conducted at the University of Guelph NMR Center. The 4,4-dimethyl-4-silapentane-1-sulfonate (DSS) was used as the internal standard. Briefly, sterile-filtered extracts (540 µL) were mixed with 60 µL Chenomx IS-2 stock solution (4.94 mM DSS-d_6_; Chenomx, AB, Canada) to achieve a final DSS concentration of 0.494 mM. A 560 µL aliquot was transferred to a 5 mm NMR tube for analysis. Spectra were acquired at 298 ± 1 K on a Bruker AVANCE III spectrometer equipped with a 5 mm TCI cryoprobe, using a noesypr1d pulse sequence with 128 scans, a 2 s relaxation delay with water presaturation, and a 3 s acquisition period. Data were processed in Chenomx NMR Suite v10.0 with 0.2 Hz line broadening, manual phase and baseline correction, automated shim correction, and automated CSI calibration. Because 2-methylbutyrate is not included in the Chenomx library, it was manually added using a reference spectrum from a pure standard, as per the Chenomx user manual. Metabolite levels were normalized to total protein content, measured using the Pierce BCA Protein Assay Kit (Thermo Scientific, Cat. No. LSG23227).

### Reverse transcription quantitative real-time PCR

Overnight cultures of *hcpR*::Tn and WT strains were grown in BHI broth to OD_600_ = 0.2 and then treated with 0.0625 mM NO donor or DMSO control for 30 minutes. Cells were harvested by centrifugation at 4,000 rpm for 5 minutes, and pellets were resuspended in 1 mL of RNAprotect Bacterial Reagent (Qiagen, Cat. No. 76506) to preserve RNA integrity. Pellets were stored for RNA extraction following a final centrifugation. Total RNA was extracted using the HiPure Total RNA Mini Kit (GeneBio Systems, Canada, Cat. No. R401102) and treated with TURBO DNase (Thermo Fisher, Cat. No. AM1907) to eliminate genomic DNA. cDNA synthesis was performed using qScript cDNA SuperMix (Quantabio, Cat. No. 95048-100), and qPCR was conducted with PerfeCTa SYBR Green SuperMix (Quantabio, Cat. No. 95074-012) on a QuantStudio 6 Flex Real-Time PCR System. Gene expression was normalized to 16S rRNA gene and fold change calculated using the 2^−ΔΔCt^ method (52). Primers used are listed in Table S8 (26, 53, 54).

### RNA sequencing

WT and *hcpR*::Tn strains were treated with the NO donor or DMSO control (unstressed condition) for 30 minutes as described above. RNA extraction and purification were done as already described for qPCR. Samples were sent to The Center for Applied Genomics (Toronto) for sequencing. Libraries were prepared using the Illumina Stranded Total RNA Prep kit with rRNA depletion using the Ribo-Zero Plus Microbiome kit. Paired-end sequencing was performed on the NovaSeq X system using a 10B flow cell to generate 150 bp reads. RNA-seq analysis was performed using the previously described methods (26). Sequencing data were uploaded to the Galaxy platform (https://usegalaxy.org/), quality-checked with FastQC, and trimmed using Trim Galore. Reads were mapped to the *C. difficile* R20291 genome (accession no. FN545816.1) using BWA-MEM2. Read counts were obtained with htseq-count, and differential expression analysis was performed using edgeR in Degust (http://degust.erc.monash.edu/) with cutoffs of log_2_ fold change ≥ 1 and false discovery rate (FDR) ≤ 0.01. Because *hcpR* is inactivated in the *hcpR*::Tn strain, residual reads mapping to its sequence were detected by RNA-seq. Although *hcpR* appeared in the DEG output, it was excluded from downstream analyses. A volcano plot was generated using VolcaNoseR (https://huygens.science.uva.nl/VolcaNoseR/), and a heatmap was created using Morpheus (https://software.broadinstitute.org/morpheus/). GSEA with KEGG pathway was performed using ClusterProfiler, with significance defined as adjusted *P* < 0.05 and gene sets classified as upregulated (NES > 0) or downregulated (NES < 0) (55). A relaxed threshold of *q* ≤ 0.25 was also used. KEGG metabolic pathway analysis of the DEGs was performed using KEGG Mapper via KofamKOALA.

## Data Availability

Raw RNA-seq data are available in the NCBI database under accession number PRJNA1204944.

## SUPPLEMENTAL MATERIAL

Fig. S1 aem.01988-25-s0001.pdf Growth of a CRISPRi *nimB* knockdown (*nimB*-kd) strain under increasing concentrations of the NO donor DETA/NO.

Supplemental tables aem.01988-25-s0002.xlsx Tables S1 to S8.

ASM does not own the copyrights to Supplemental Material that may be linked to, or accessed through, an article. The authors have granted ASM a non-exclusive, world-wide license to publish the Supplemental Material files. Please contact the corresponding author directly for reuse.
