# Supplementary material for: The critical role of hcpR in regulating nitrosative stress defense in Clostridioides difficile
Source: Appl Environ Microbiol. 2026 Jan 26;92(2):e01988-25. doi: 10.1128/aem.01988-25 (PMC12915319; doi:10.1128/aem.01988-25)
Supplement: Fig. S1 — Growth of a CRISPRi nimB knockdown (nimB-kd) strain under increasing concentrations of the NO donor DETA/NO. [file aem.01988-25-s0001.pdf]

Supplementary figure

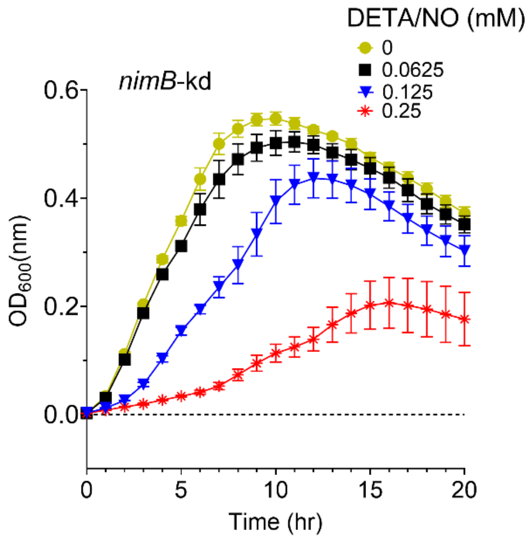

Fig. S1. Growth of a CRISPRi *nimB* knockdown (*nimB*-kd) strain under increasing concentrations of the NO donor DETA/NO. Within the NO concentration range used in this study, *nimB* knockdown did not alter sensitivity to nitrosative stress compared to the DMSO control without the NO donor. Data are presented as mean ± SEM from three biological replicates.
